# CT perfusion-guided administration of IV milrinone is associated with a reduction in delayed cerebral infarction after subarachnoid hemorrhage

**DOI:** 10.1038/s41598-024-65706-w

**Published:** 2024-06-27

**Authors:** Vivien Szabo, Sarah Baccialone, Florentin Kucharczak, Cyril Dargazanli, Oceane Garnier, Frederique Pavillard, Nicolas Molinari, Vincent Costalat, Pierre-Francois Perrigault, Kevin Chalard

**Affiliations:** 1 Department of Critical Care Medicine and Anesthesiology (DAR GDC), Gui de Chauliac University Hospital of Montpellier, Montpellier, France; 2 IGF, Univ. Montpellier, CNRS UMR5203, Inserm U1191, Montpellier, France; 3 Department of Biostatistics, Clinical Epidemiology, Public Health and Innovation in Methodology (BESPIM), Nîmes University Hospital Center, Univ. Montpellier, Nimes, France; 4 Department of Nuclear Medicine, Gui de Chauliac University Hospital of Montpellier, University of Montpellier, Montpellier, France; 5 Department of Neuroradiology, Gui de Chauliac University Hospital of Montpellier, Montpellier, France; 6 Epidemiology and Clinical Research Department, University Hospital of Montpellier, Montpellier, France; 7 IMAG, Univ Montpellier, CNRS, CHU Montpellier, Montpellier, France

**Keywords:** Medical research, Cerebrovascular disorders

## Abstract

Delayed cerebral ischemia (DCI) after aneurysmal subarachnoid haemorrhage (aSAH) is a singular pathological entity necessitating early diagnostic approaches and both prophylactic and curative interventions. This retrospective before-after study investigates the effects of a management strategy integrating perfusion computed tomography (CTP), vigilant clinical monitoring and standardized systemic administration of milrinone on the occurrence of delayed cerebral infarction (DCIn). The ${\text{"before"}}$ period included 277 patients, and the ${\text{"after"}}$ one 453. There was a higher prevalence of Modified Fisher score III/IV and more frequent diagnosis of vasospasm in the ${\text{"after"}}$ period. Conversely, the occurrence of DCIn was reduced with the ${\text{"after"}}$ management strategy (adjusted OR 0.48, 95% CI [0.26; 0.84]). Notably, delayed ischemic neurologic deficits were less prevalent at the time of vasospasm diagnosis (24 vs 11%, $p=0.001$), suggesting that CTP facilitated early detection. In patients diagnosed with vasospasm, intravenous milrinone was more frequently administered (80 vs 54%, $p<0.001$) and associated with superior hemodynamics. The present study from a large cohort of aSAH patients suggests, for one part, the interest of CTP in early diagnosis of vasospasm and DCI, and for the other the efficacy of CT perfusion-guided systemic administration of milrinone in both preventing and treating DCIn.

## Introduction

Aneurysmal subarachnoid hemorrhage (aSAH) presents with an incidence of 8–10/100,000 inhabitants/year^1–3^. It most often affects women, with a female to male ratio of 3/2, and a mean age of occurrence of 55–62 years^3–5^. During the last couple of decades, mortality rates showed no tendency to overall improvement^2,6^, and nowadays outcome remains poor, resulting in mortality or severe handicap in 30% of patients^4,7,8^.

In this context, delayed cerebral ischemia (DCI), a major risk factor for poor clinical outcome as frequent as in 25–30% of aSAH patients, appears to be a singular pathological entity with a time course eligible to both prophylactic and curative treatments^4,9,10^. Commonly diagnosed on computed-tomography (CT) or magnetic resonance (MR) scans when irreversible delayed cerebral infarction (DCIn) is established^11^, DCI can also be suspected early using transcranial Doppler (TCD)^12–14^ or perfusion CT (CTP) in order to diagnose vasospasm and measure its impact on cerebral perfusion before the infarct has formed^12,15–17^.

Even though those diagnostic tools are widely accepted, consensual treatments are very few^18,19^, and first consist in non-pharmacological approaches aiming at optimizing systemic and ergo cerebral hemodynamics, mainly through euvolemia^20,21^ and elevated blood pressure^22,23^. The principal drug used in prophylaxis is nimodipine, which systemic administration has been shown to improve patients outcome^24,25^.

Other less consensual treatments include direct arterial dilation via intra-arterial administration of pharmacological agents^26–28^ including milrinone^29–32^ or percutaneous transluminal angioplasty (PTA)^28,33,34^, as well as systemic intravenous (IV) administration of milrinone^5^. This last option, mainly based on inodilation, has these past few years received particular attention. Indeed, it has been repeatedly suggested that IV milrinone could be both efficient and safe for improving aSAH patients’ clinical outcome^35–37^. However, data are still sparse and come from studies of small sample sizes^31,38–40^, leading the most recent USA Guidelines for aSAH patients management to conclude that “*the role of milrinone, although promising, requires further investigation*”^5^.

In this before-after study, we aimed at assessing the effects of a new management strategy, comprising systematic CTP follow-up and the administration of IV milrinone, on the occurrence of DCIn in aSAH patients.

## Materials and methods

### Study population

This retrospective, before-after, monocentric study was conducted in an academic center in Montpellier, France. French adult patients hospitalized for aSAH confirmed by angio-CT (CTA) during two distinct periods, from 2014 to 2016 (“before”) and from 2018 to 2021 (“after”) were included.

### Management protocol

#### Strategy common to the two periods

Aneurysm exclusion was performed according to the current guidelines for aSAH management^5^. Endovascular or surgical securization procedure was collegially decided by neurosurgeons and neuroradiologists. Surgical procedures including external ventricular drainage (EVD), intracerebral hemorrhage (ICH) evacuation or decompressive craniectomy were performed when necessary. Comatose patients were sedated and assisted with mechanical ventilation to control oxygenation and ventilation (PaO_2_ $\ge$ 75 mmHg, PaCO_2_ between 35 and 40 mmHg). Body temperature was maintained between 36 and 38 $^{\circ }$C. All patients received nimodipine, administered either orally every 4 h in a total daily dose of 360 mg, or intravenously in a dose of 2 mg/h when the oral route was not available. Patients were under continuous nurse supervision and regular physician examination notably to detect neurological deterioration associated with DCI. When suspected after TCD and/or clinical examination, angiographic vasospasm and DCI were evaluated on CT or MR scans. In patients with angiographic vasospasm and/or DCI, cerebral perfusion pressure was optimized by maintaining normovolemia and inducing hypertension using norepinephrine when necessary.

#### Rescue therapy during the “before” period

Patients diagnosed with angiographic vasospasm presenting either a prolonged neurological deficit or a persistent vasospasm when unconscious were treated with intra-arterial administration of both nimodipine and milrinone. As a last resort, PTA was considered when vasospasm concerned the proximal portion of the middle cerebral artery. Most severe vasospasms, i.e. diffuse or recurrent, were treated with continuous intravenous infusion of milrinone.

#### Preventive and rescue therapies during the “after” period

**Figure 1:**
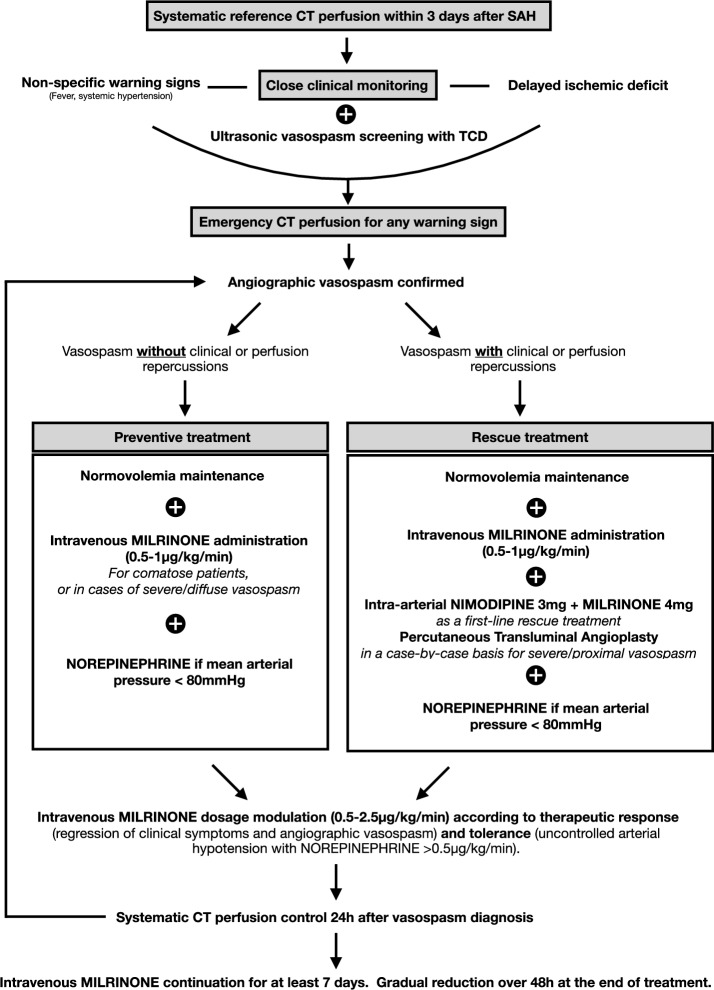
Schematic representation of practice guidelines in the “ after” period.

The “after” management strategy protocol used is depicted on Fig. 1. It was based on a systematic baseline CTP, followed by close clinical and TCD monitoring. Hypoperfusion was defined as $T_{max} > 6sec$, as calculated from the CTP sequences using automated RapidAI software (Rapid CTP for Stroke, IschemaView, Inc., Menlo Park, CA). In the following days, ultrasonic vasospasm and/or DCI suspicion led to an additional diagnostic CTP to confirm angiographic vasospasm and search for associated hypoperfusion. *Preventive treatment* (left arm of the algorithm): in comatose patients, or in cases of severe and diffuse vasospasm, IV milrinone was used even in the absence of DIND or hypoperfusion. *Rescue treatment* (right arm of the algorithm): when DIND or a hypoperfusion was present, IV milrinone was administered and direct arterial dilation was also performed using at least pharmacological agents, and through PTA after case-by-case evaluation. Raising blood pressure was not the primary goal of the “after” management, the emphasis was instead on inodilation. Any occurrence of hypotension was addressed by adding IV norepinephrine to ensure a mean arterial blood pressure (MAP) above 80 mmHg. When IV milrinone was poorly tolerated in this regard, IV milrinone dosage was reduced or administration was stopped. Clinical monitoring and routine follow-up CTP scans were performed to adjust the dose of milrinone.

### Data collection

The following data were recorded for every patient: age, sex, Glasgow Coma Scale (GCS) at admission, presence of ICH, aneurysm size and location, modified Fisher score, World Federation of Neurosurgeons Score (WFNS), type of aneurysm securing procedure and EVD placement.

Vasospasm occurrence was defined as a reduction in arteries diameter of at least one-third compared to baseline caliber, measured on either CTA or digital subtraction angiography (DSA) at any time.

Delayed ischemic neurologic deficit (DIND) was defined as a delayed clinical deterioration due to delayed cerebral ischemia (i.e. focal neurological impairment or a decrease of at least 2 points on the GCS that lasts for at least 1 h that cannot be attributed to another cause).

Indication, administration, timing and duration of IV milrinone, IA vasodilators, and PTA were recorded. For a 5-day period divided into 6-h subperiods starting at the time of vasospasm diagnosis, minimal mean arterial pressure, maximum norepinephrine dose, and maximal IV milrinone dose were noted. Mechanical ventilation, ICU and hospital stays durations, as well as vital status at hospital discharge were recorded.

### Outcome

Delayed cerebral infarction (DCIn) was defined as the presence of a cerebral infarction 1) *de novo* on the last available CT or MR scan at the time of discharge from the ICU, i.e. excluding early brain injuries (EBI) already present in either CT or MR scans performed within the first 72 h following aneurysm rupture, 2) not attributable to any other cause.

### Methodological compliance and ethics

This observational study was performed according to the STROBE guidelines. The study was validated by Montpellier ethics committee (IRB-MTP_2023_01_202301324) in accordance with French regulation and registered on the Health Data Hub study registration platform (N$^{\circ }$ F20230217101049). Since there was no invasive procedure in addition to French ICUs standard of care, patients or their family were informed and could opt out of the study, according to French law^41^.

### Statistical analysis

The statistical analyses carried out in this article are of two types. First, descriptive statistics were used to compare the variables of interest according to the groups “before” (2014-2016) and “after” (2018-2021) practices modifications, throughout the entirety of the study’s sample population, as well as within the subset of individuals who experienced vasospasm. Continuous variables were presented as means with their associated standard deviations (SD) and categorical variables with their frequencies and associated percentage. Distributions between groups were compared using the Wilcoxon Rank-Sum test to compare continuous variables and Pearson’s Chi-Square test for categorical variables (or exact Fisher test for small sample size with $N<5$). Computed p-values were given in each table.

Second, to account for imbalances between groups in confounding factors, we used a propensity score to adjust for between-group differences through a matching procedure. The propensity score was calculated using logistic regression to balance the distribution of covariates between groups of patients before and after practices modifications, allowing to reduce potential bias in the estimates of the treatment effect^42^. The following covariates were included in the score because of their clinical relevance : age, sex, modified Fisher score, WFNS, aneurysm location, aneurysm exclusion, intracerebral hemorrhage and EVD. The initial sample included two pools of patients, totalizing 730 observations. Using the optimal 1:1 pair matching method, the balanced sample consisted of 554 matched pairs. Validity of the produced score and balance of covariates were assessed using standardized mean differences, propensity score histograms and with statistical comparison of the “before” and “after” populations after matching. Inverse probability of treatment weighting (IPTW) based on the propensity score was used in a logistic regression model to explore the effect of the changes in practice on DCIn occurrence. Unadjusted odds-ratio and IPTW adjusted odds-ratio (OR) are presented with their 95% confidence interval, as well as the corresponding p-value.

All statistical analyses were conducted using R software, version 4.2.1. A p-value threshold of less than 0.05 was adopted for all tests for statistical significance.

### Ethics approval

This study was validated by Montpellier ethics committee (IRB-MTP_2023_01_202301324) in accordance with French regulation and registered on the Health Data Hub study registration platform (N$^{\circ }$ F20230217101049).

### Consent to participate

Since there was no invasive procedure in addition to French ICUs standard of care, patients or their family were informed and could opt out of the study.

## Results

The “before” period spanned from 2014 to 2016 and enrolled 277 patients, the “after” one from 2018 to 2021 and included 453 patients. Six patients were excluded because of refusal. Patients and aSAH characteristics in both periods are presented in Table 1.

**Table 1:** Descriptive table for groups “before” and “after” practices modifications

	Whole population			After matching		
	Before, N = 277$^{{1}}$	After, N = 453$^{{1}}$	p-value	Before, N = 277$^{{2}}$	After, N = 277$^{{2}}$	p-value
Age*	55.22 (13.38)	56.21 (12.19)	0.2$^{{3}}$	55.22 (13.38)	55.58 (12.36)	0.6$^{{3}}$
Gender*			0.3$^{{4}}$			0.2$^{{4}}$
Female	184 (66%)	283 (62%)		184 (66%)	170 (61%)	
Male	93 (34%)	170 (38%)		93 (34%)	107 (39%)	
Modified Fisher score*			$<0.001^{{4}}$			$>0.9^{{4}}$
I/II	101 (36%)	109 (24%)		101 (36%)	102 (37%)	
III/IV	176 (64%)	344 (76%)		176 (64%)	175 (63%)	
WFNS*			0.12$^{{4}}$			0.8$^{{4}}$
I/II	163 (59%)	240 (53%)		163 (59%)	160 (58%)	
III/IV/V	114 (41%)	213 (47%)		114 (41%)	117 (42%)	
Aneurysm localisation*			0.8$^{{4}}$			$>0.9^{{4}}$
ACA/Acom	104 (38%)	177 (39%)		104 (38%)	102 (37%)	
IC/Pcom	67 (24%)	108 (24%)		67 (24%)	63 (23%)	
MCA	63 (23%)	94 (21%)		63 (23%)	68 (25%)	
Other localization	8 (2.9%)	21 (4.6%)		8 (2.9%)	9 (3.2%)	
Vertebrobasilar territory	35 (13%)	53 (12%)		35 (13%)	35 (13%)	
Aneurysm exclusion*			0.2$^{{4}}$			$>0.9^{{4}}$
No treatment	12 (4.3%)	34 (7.5%)		12 (4.3%)	12 (4.3%)	
Clipping	31 (11%)	49 (11%)		31 (11%)	31 (11%)	
Coiling	234 (84%)	370 (82%)		234 (84%)	234 (84%)	
Aneurysm size (cm)			0.2$^{{4}}$			0.067$^{{4}}$
$\le$5	64 (30%)	160 (37%)		64 (30%)	104 (39%)	
]5–10[	100 (47%)	194 (45%)		100 (47%)	119 (45%)	
$\ge$10	47 (22%)	78 (18%)		47 (22%)	42 (16%)	
(Missing data)	66	21		66	12	
Rebleeding	35 (13%)	63 (14%)	0.6$^{{4}}$	35 (13%)	37 (13%)	0.8$^{{4}}$
ICH*	81 (29%)	147 (32%)	0.4$^{{4}}$	81 (29%)	79 (29%)	0.9$^{{4}}$
Early brain injury	82 (30%)	144 (32%)	0.5$^{{4}}$	82 (30%)	86 (31%)	0.7$^{{4}}$
EVD	120 (43%)	214 (47%)	0.3$^{{4}}$	120 (43%)	125 (45%)	0.7$^{{4}}$
Vasospasm	118 (43%)	259 (57%)	<**0.001**$^{{4}}$	118 (43%)	154 (56%)	**0.002**$^{{4}}$
Length of MV, days	18.70 (17.56)	15.01 (15.72)	**0.015**$^{{3}}$	18.70 (17.56)	15.31 (16.51)	**0.032**$^{{3}}$
Death			0.2$^{{4}}$			$>0.9^{{4}}$
No	233 (84%)	363 (80%)		233 (84%)	232 (84%)	
Yes	44 (16%)	90 (20%)		44 (16%)	45 (16%)	
Hospital LOS, days	29.25 (26.03)	29.28 (25.12)	0.4$^{{3}}$	29.25 (26.03)	29.73 (26.34)	0.4$^{{3}}$
ICU LOS, days	26.18 (24.07)	19.55 (19.14)	**0.008**$^{{3}}$	26.18 (24.07)	20.29 (19.94)	**0.040**$^{{3}}$

* WFNS* World Federation of Neurological Surgeons,* ACA* Anterior cerebral artery,* Acom* Anterior communicating artery,* IC* Internal carotid,* Pcom* Posterior communicating artery,* MCA* Middle cerebral artery,* ICH* Intracerebral hemorrhage,* EVD* External ventricular drainage,* MV* Mechanical ventilation,* LOS* Length of stay,* ICU* Intensive Care Unit. $^{1}$ Mean (SD); n (%), $^{2}$ Wilcoxon rank sum test, $^{3}$ Pearson’s Chi-squared test. $^{4}$ Fisher exact test. Variables denoted with a * were included in the matching procedure.

Significant values are in bold.

Patients age and sex ratio were similar, WFNS and aneurysm characteristics were comparable, and EBI was as frequent in the two groups. There was no difference regarding aneurysm treatment (i.e. embolization vs surgery). At the time of admission, only the modified Fisher score differed between groups, being more frequently III/IV during the “after” period. After adjustment, the baseline variables distributions are well balanced between the two groups. Besides, vasospasm was more frequently diagnosed during the “after” than in the “before” period (56% vs 43% respectively, $p=0.002$). Length of mechanical ventilation and ICU length of stay were reduced in the “after” period. All-cause mortality was similar between the two periods.

Table 2 presents the OR for DCIn in the “after” compared to the “before” period, both unadjusted (0.64, IC95=[0.39; 1.01] and adjusted (0.48, IC95= [0.26; 0.84]), showing a reduction in DCIn occurrence when the new management strategy was established.

**Table 2:** Effect of change in practice on DCIn occurrence after population matching (N=544). OR=Odds ratio, CI=Confidence interval.

	Without adjustment			Adjusted		
DCIn	OR	95% CI	p-value	OR	95% CI	p-value
Period						
Before	–	–		–	–	
After	0.63	0.39, 1.01	0.055	0.48	0.26, 0.84	**0.013**

In order to explore the effective management of vasospasm and DCI in the two periods, the remaining of the study focuses on the subpopulation of patients diagnosed with vasospasm, before statistical matching. This subpopulation’s characteristics are presented in Table 3. Patients and aneurysm features were all similar in the two periods of time. However, DCIn and DIND, both at the time of vasospasm diagnosis and at any time during the stay, were less frequent in the “after” than in the “before” period (15 vs 31% $p<0.001$, 11 vs 24% $p=0.001$, 15 vs 26% $p=0.013$ respectively).

**Table 3:** Descriptive table of the vasospasm subpopulation (N=377)

	Before, N = 118$^{{1}}$	After, N = 259$^{{1}}$	p-value
Age	54.55 (12.52)	55.66 (11.55)	0.4$^{{2}}$
Sex			0.4$^{{3}}$
Female	81 (69%)	165 (64%)	
Male	37 (31%)	94 (36%)	
Modified Fisher score			0.2$^{{3}}$
I/II	27 (23%)	45 (17%)	
III/IV	91 (77%)	214 (83%)	
WFNS			$>0.9^{{3}}$
I/II	60 (51%)	131 (51%)	
III/IV/V	58 (49%)	128 (49%)	
Aneurysm localisation			0.9$^{{4}}$
ACA/Acom	48 (41%)	104 (40%)	
IC/Pcom	30 (25%)	64 (25%)	
MCA	28 (24%)	55 (21%)	
Other localization	2 (2%)	8 (3%)	
Vertebrobasilar territory	10 (8%)	28 (11%)	
Aneurysm exclusion			0.7$^{{4}}$
No treatment	1 (1%)	1 (1%)	
Clipping	12 (10%)	30 (11%)	
Coiling	105 (89%)	228 (88%)	
Intracerebral hemorrhage	44 (37%)	88 (34%)	0.5$^{{3}}$
External ventricular drainage	65 (55%)	162 (63%)	0.2$^{{3}}$
Delayed cerebral infarction (DCIn)	37 (31%)	40 (15%)	<**0.001**$^{{3}}$
DIND at vasospasm diagnosis	28 (24%)	28 (11%)	**0.001**$^{{3}}$
DIND during the stay	31 (26%)	40 (15%)	**0.013**$^{{3}}$

* WFNS* World Federation of Neurological Surgeons,* ACA* Anterior cerebral artery,* Acom* Anterior communicating artery,* IC* Internal carotid,* Pcom* Posterior communicating artery,* MCA* Middle cerebral artery,* DIND* Delayed ischemic neurologic deficit. $^{1}$ Mean (SD); n (%), $^{2}$ Wilcoxon rank sum test, $^{3}$ Pearson’s Chi-squared test, $^{4}$ Fisher’s exact test.

Significant values are in bold.

The treatments administered in patients with vasospasm are shown in Table 4. First, indication for treatment was different in the two periods. Indeed, DIND and DCIn were less frequently a motive in the “after” period, being only 8 versus 21% and 1 versus 9% respectively ($p<0.001$). Accordingly, vasodilation was more frequently undertaken as a preventive treatment in the “after” period (71 vs 51%). Second, IV milrinone was in fact more frequently administered in the “after” period (80 vs 54%, $p<0.001$), although at the same mean dose (0.84 vs 0.83$\mu$g/kg/min, $p=0.606$). IA administration of vasodilators was similar in the two periods, while PTA was less frequently performed in the “after” one (6.6 vs 16%, $p=0.003$).

**Table 4:** Profile of treatments used in the vasospasm subpopulation (N=377).

	Before, N = 118$^{{1}}$	After, N = 259$^{{1}}$	p-value
Untreated vasospasm	39 (33%)	51 (20%)	<**0.005**$^{{2}}$
IV milrinone administration	64 (54%)	208 (80%)	<**0.001**$^{{2}}$
IA milrinone administration	49 (42%)	83 (32%)	0.074$^{{2}}$
IA nimodipine administration	50 (42%)	83 (32%)	0.052$^{{2}}$
PTA	19 (16%)	17 (6.6%)	**0.003**$^{{2}}$
Indication for vasospasm treatment			<**0.001**$^{{3}}$
Delayed ischemic neurologic deficit (DIND)	17 (21%)	16 (8%)	
Hypoperfusion on perfusion CT	15 (19%)	44 (21%)	
Delayed cerebral infarction (DCIn)	7 (9%)	1 (1%)	
Prevention	40 (51%)	147 (70%)	

* IV* Intravenous,* IA* Intra-arterial,* PTA* Percutaneous transluminal angioplasty. $^{1}$ n (%), $^{2}$ Pearson’s Chi-squared test, $^{3}$ Fisher’s exact test.

Significant values are in bold.

**Figure 2:**
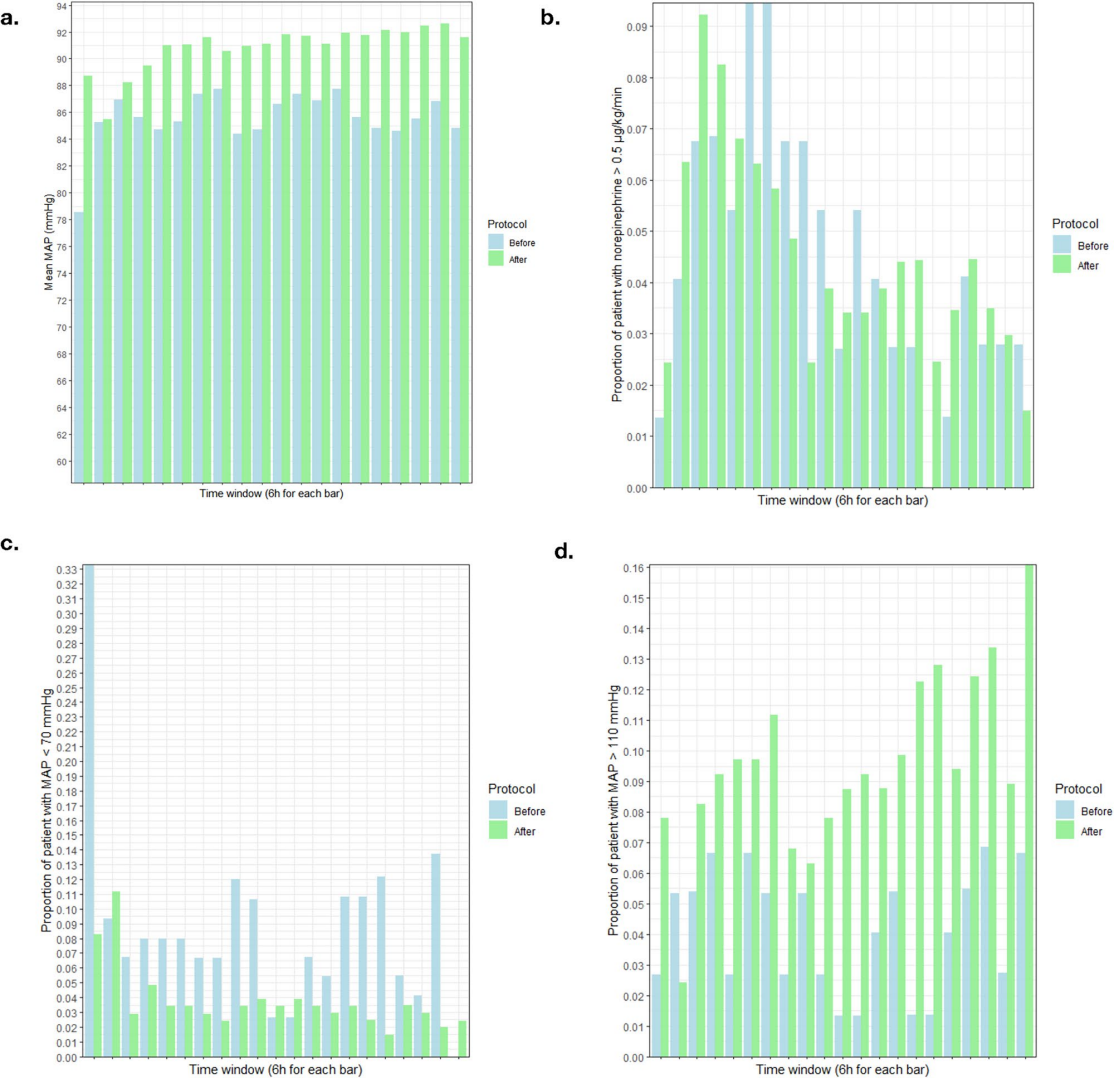
Indicators of systemic hemodynamics in the 5-day period following vasospasm diagnosis, in the “before”(blue) and “after”(green) groups. (**a**) Mean arterial blood pressure (MAP). (**b**) Proportion of patients in whom norepinephrine exceeded 0.5$\mu$g/kg/mn. (**c**) Proportion of patients showing a MAP lower than 70 mmHg. (**d**) Proportion of patients showing a MAP higher than 110 mmHg.

Finally, we report indicators of systemic hemodynamics in Fig. 2. In the “after” period, mean MAP was slightly higher (91 vs 86 mmHg, $p<0.001$, see Fig. 2a), and less patients presented with MAP<70 mmHg and more with MAP>110 mmHg (Fig. 2c, d). In order to achieve and maintain elevated blood pressure, no more patients required a dose of norepinephrine higher than 0.5$\mu$g/kg/mn in the “after” period (Fig. 2b), even though the mean dose was slightly higher (0.35 vs 0.28$\mu$g/kg/min, $p<0.001$).

## Discussion

This before-after study shows a reduction in delayed cerebral infarction associated with a management strategy based on systematic early CTP guiding systemic administration of milrinone in aSAH patients.

Patients and aSAH characteristics were comparable in the two periods, except for high Fisher scores and vasospasm diagnosis, both more frequent in the “after” period. It is possible that vasospasm was more frequently diagnosed because of the management strategy. Alternately, it could actually have been more frequent, which would be consistent with the distribution of Fisher scores^43^. In either case, these findings can only strengthen the conclusions on the positive effects of the new strategy on the outcome.

In details, this strategy led to early asymptomatic vasospasm diagnosis, allowing neurological deficit and established infarction to be less frequent at the time of treatment initiation. Additionally, intravenous milrinone reduced the need for PTA, and was accompanied by superior systemic hemodynamics.

Of course, the nature of the study leads to limitations, mainly concerning long-term neurological outcome, the record of which was not available. All the same, we present real-world data from unselected patients together with detailed management description, such that our bundle of care can easily be reproduced or adapted in other centers to achieve the low incidence of DCIn seen in our cohort.

There are several putative explanations for the reduced occurrence of DCIn with the new strategy. First, early asymptomatic diagnosis due to systematic and following CTP could have triggered early treatment, plausibly associated with regression or compensation of pathological mechanisms. This interest of CTP in the early phase of delayed ischemia has already been suggested in previous studies^16,44,45^. However, the clinical impact of early diagnosis using this method had not yet been conclusively demonstrated. We report here arguments in favor of this strategy, adding data from critically ill patients, lacking from the literature^44^.

Second, the proactive approach combining first-line milrinone with limited doses of norepinephrine could maximize the positive effects of milrinone on cerebral hemodynamics by prioritizing cerebral vasodilation over the achievement of systemic arterial hypertension, even though this strategy is still a matter of debate^39,46,47^. It is suggested that milrinone systemic effects on hemodynamics, namely increased cardiac output and increased venous return, can reasonably improve brain perfusion when associated with cerebral arteries dilation^35,48^. Indeed, the Lassen curve could be shifted upwards and rightwards after milrinone administration, leading to a greater cerebral blood flow for a given cerebral perfusion pressure^35^.

Third, systemic administration of milrinone can have several other consequences on disease progression. For instance, pharmacological vasodilation of cerebral arteries is with this approach not limited to large vessels diagnosed with vasospasm. It is likely that small arteries and arterioles, at the core of recent pathophysiological theories for DCI and not accessible to PTA, benefit from milrinone effects on smooth muscle cells^49–52^. Another postulated mechanism involves anti-inflammatory properties of milrinone through inhibition of cytokines production^35^, since inflammation is also suspected to play a role in DCI development^53^. For instance, perivascular macrophage have been shown to participate in microvascular spasms in experimental models, and could therefore be a cellular target for milrinone non-vascular effects^54^.

## Conclusion

Even though the underlying causes for the observed effects remain mainly speculative, the present study shows data from a large cohort of 730 patient suggesting, for one part, the interest of CTP in early diagnosis of vasospasm and DCI, and for the other the efficacy of CT perfusion-guided systemic administration of milrinone in prevention and treatment of DCIn.

## Data Availability

The datasets used and analysed during the current study are available from the corresponding author on reasonable request.
